# Statistical power and utility of meta-analysis methods for cross-phenotype genome-wide association studies

**DOI:** 10.1371/journal.pone.0193256

**Published:** 2018-03-01

**Authors:** Zhaozhong Zhu, Verneri Anttila, Jordan W. Smoller, Phil H. Lee

**Affiliations:** 1 Center for Genomic Medicine, Massachusetts General Hospital and Harvard Medical School, Boston, Massachusetts, United States of America; 2 Program in Quantitative Genomics, Harvard T.H. Chan School of Public Health, Boston, Massachusetts, United States of America; 3 Stanley Center for Psychiatric Research, Broad Institute of MIT and Harvard, Cambridge, Massachusetts, United States of America; Indiana University Bloomington, UNITED STATES

## Abstract

Advances in recent genome wide association studies (GWAS) suggest that pleiotropic effects on human complex traits are widespread. A number of classic and recent meta-analysis methods have been used to identify genetic loci with pleiotropic effects, but the overall performance of these methods is not well understood. In this work, we use extensive simulations and case studies of GWAS datasets to investigate the power and type-I error rates of ten meta-analysis methods. We specifically focus on three conditions commonly encountered in the studies of multiple traits: (1) extensive heterogeneity of genetic effects; (2) characterization of trait-specific association; and (3) inflated correlation of GWAS due to overlapping samples. Although the statistical power is highly variable under distinct study conditions, we found the superior power of several methods under diverse heterogeneity. In particular, classic fixed-effects model showed surprisingly good performance when a variant is associated with more than a half of study traits. As the number of traits with null effects increases, ASSET performed the best along with competitive specificity and sensitivity. With opposite directional effects, CPASSOC featured the first-rate power. However, caution is advised when using CPASSOC for studying genetically correlated traits with overlapping samples. We conclude with a discussion of unresolved issues and directions for future research.

## Introduction

Pleiotropy refers to a biological phenomenon where a single variant or a gene affects multiple phenotypes.[1] In recent years, a startling level of genome-wide genetic correlation has been revealed between various complex traits and disorders.[2–5] Moreover, a growing number of genetic loci have shown pleiotropic effects on multiple, sometimes seemingly distinct traits,[6] providing an intriguing opportunity to enhance our understanding of the shared genetic mechanisms.[7–11] The identification, characterization, and potential clinical translation of pleiotropic genetic effects present immense opportunities for genomic medicine, of which the major focus includes the development of new drugs and therapeutic targets with broad efficacy, while minimizing the unexpected side effects.

To identify genetic variants with pleiotropic effects, cross-phenotype genome-wide association studies (GWAS) have employed a range of classic and recently developed meta-analysis methods. Typically, summary statistics of distinct but potentially related traits are combined in a meta-analysis framework to detect specific loci with shared association. Such univariate approaches do not require access to individual-level genotype data, and thus are readily applicable to existing GWAS results. Combining results across studies of different traits can also improve the power of detecting modest cross-phenotype genetic effects, which may not reach genome-wide significance for any single trait.

Cross-phenotype GWAS however incur several unique challenges compared to a traditional meta-analysis of a single trait. First, the biological impact of a shared causal variant often varies among different traits,[12,13] yielding additional heterogeneity to effect size. Second, genetic variants at a particular locus may affect only a subset of study traits;[14–16] for instance, in a recent GWAS meta-analysis of three cancers, two risk loci, *SMC2* and *RCCD1*, showed association with breast and ovarian cancer but no effect on prostate cancer.[17] Some genomic loci have even showed antagonistic effects in which the same allele appears to increase the risk of one disease while protecting against another disease.[18–20] Distinguishing genuine heterogeneous effects from statistical noise is a nontrivial task especially when multiple traits of different power and study designs are evaluated together. Another unique challenge of using meta-analysis in cross-phenotype GWAS is that currently available methods do not formally test pleiotropic effects. Instead, a null hypothesis is set up such that a target variant is associated with none of the study traits. Significant meta-analysis results thus may arise even when a single trait drives the association. Most meta-analysis methods however provide statistical evidence for overall association, without indicating specific traits that drive the pleiotropic signal. Finally, it is common in the studies of related traits to encounter the use of shared controls or even overlapping cases of distinct diseases. Such overlap induces artificial correlation among association test statistics, resulting in inflated false positive findings.[16] A series of new meta-analysis methods were introduced to address some of these challenges, but major questions related to their behavior and performance remain unanswered.

The aim of this study is to evaluate the performance of seven recently developed meta-analysis methods in various contexts of cross-phenotype GWAS: (1) One-sided Association analysis based on SubSETs (ASSET1)[15], (2) Two-sided Association analysis based on SubSETs (ASSET2)[15], (3) Binary Effect (BE)[21], (4) Han and Eskin Random Effect meta-analysis (HE-REMA)[22], (5) Cross Phenotype Association (CPASSOC)[16], (6) Weighted Inverse Chi-Sqaure (WICS)[23], and (7) Cross Phenotype Meta-Analysis (CPMA)[14]. These methods build upon three classic meta-analysis approaches, which are also included in this study as base models: (1) the fixed-effects meta-analysis (FEMA) model[24], (2) the random-effects meta-analysis (REMA) model[25], and (3) Fisher’s method (Fisher)[26]. Table 1 summarizes the major characteristics of the ten meta-analysis methods we evaluate in this study. Using a series of simulations and case studies of GWAS data, we investigate the power and type-I error rates of these methods and illustrate how their performance changes under various scenarios particularly relevant to cross-phenotype studies. We conclude this study with a discussion of methodological issues that demand further improvements.

**Table 1:** Summary of ten meta-analysis methods evaluated in this study.

Category	Methods	Effect Size Estimation	Control of Shared Subjects	Agnostic to Effect Direction	Prediction of Associated Trait	Reference	Software
Fixed-Effects	FEMA	yes	no	no	no	Greenland et al.[24]	PLINK
	ASSET1	yes	yes	no	yes	Bhattacharjee et al.[15]	ASSET
	ASSET2	yes	yes	yes	yes	Bhattacharjee et al.[15]	ASSET
	BE	no	no	no	yes	Han et al.[21]	METASOFT
	CPASSOC	no	yes	yes	no	Zhu et al.[16]	R code
Random-Effects	REMA	yes	no	no	no	DerSimonian et al.[25]	PLINK
	HE-REMA	yes	no	no	no	Han et al.[22]	METASOFT
P-value	Fisher	no	no	yes	no	Fisher[26]	R code
	WICS	no	yes	yes	no	Zaykin et al.[23]	R code
	CPMA	no	no	yes	no	Cotsapas et al.[14]	R code

## Methods

### Cross-phenotype meta-analysis methods

We examined the performance of ten published meta-analysis methods: (1) FEMA; (2) ASSET1; (3) ASSET2; (4) BE; (5) CPASSOC; (6) REMA; (7) HE-REMA; (8) Fisher; (9) WICS; (10) CPMA. We summarized the major characteristics of ten meta-analysis methods in Table 1. For further details of the methods, we refer to S1 Note and original publications[14–16,21–26].

### Simulation studies to assess power and type I error rates

We generated 10,000 sets of association summary statistics for *K* study traits (*K* = 5, 8, 10), assuming causal SNPs in Hardy-Weinberg equilibrium with the population minor allele frequency (MAF) *f* (*f* = 0.1). We refer to the number of traits with non-null effects as *T*_*a*_ (2 ≤ *T*_*a*_ ≤ *K*). To examine a list of scenarios with various heterogeneous effects, we tested three types of effect size distributions: (1) normal distribution, (2) bimodal normal distribution, and (3) uniform distribution. For the normal distribution, the effect size β_i_ was selected from *N* (ln(*μ*), (*k*ln(*μ*))^2^) where *μ* = 1.1 and *k* = 0.5 (*i* ∈ {1,2,…,*K*}). When a trait was not associated with an SNP, the effect size β_i_ was drawn from *N* (0, (*k*ln(*μ*))^2^) where *k* = 0.25. For traits with opposite directional effects, we assigned a negative sign once the effect size β_i_ is randomly selected for the trait. For the scenario of bimodal normal distribution, the effect size β_i_ of each trait was generated from one of two independent normal distributions, *N* (0.5ln(*μ*), (*k*ln(*μ*))^2^) and *N* (1.5ln(*μ*), (*k*ln(*μ*))^2^), with equal probabilities. Under the uniform distribution, we chose the effect size β in the range of 0 to 2ln(*μ*). We assumed an equal number of subjects for K traits, setting *N* cases and *N* controls (*N* = 1,000). Given a randomly selected effect size and *N*, the MAF of a variant in cases was calculated assuming the control MAF *f*, and the 1-degree-of-freedom chi-square test was conducted for assessing association with each trait. This procedure was repeated 10,000 times for *K* traits under each setting. We repeated the same procedure assuming the odds ratios (ORs) *μ* of 1.2.

The empirical power of each method was calculated as the proportion of the simulation runs using non-null effects where the meta-analysis *p*-value was significant at a designated α level (α = 0.05). The type I error rate was estimated as the proportion of the simulation runs showing significant *p*-value when the null hypothesis was true at α = 0.05, 0.01, and 0.001.

### Prediction of trait-specific associations

For the methods ASSET1, ASSET2, CPASSOC, and BE we calculated the sensitivity and the specificity of each method under the same simulation settings we described in the previous section. The ASSET and CPASSOC output the predicted list of associated traits. We calculated the average proportion of detected associated traits (i.e., sensitivity) and the average proportion of discarded null traits (i.e., specificity) using the predicted lists. The BE method outputs the posterior probability *m*-value for each trait instead of binary classification of traits. We thus calculated the sensitivity and the specificity of the BE method using the *m*-value thresholds as suggested in the original publication[21]: the minimum *m*-value of 0.9 for non-null effects and the maximum of 0.1 for null effects. This prediction mode was referred to as BE_m.0.9/0.1_. This classification however left many traits indeterminate. For comparison with other methods, we thus used the *m* value of 0.5 to divide all traits into either non-null (*m*>0.5) or null (*m*≤0.5) effects. We referred to this classification mode as BE_m.0.5/0.5_.

In addition to simulation, we empirically examined the performance of ASSET, CPASSOC, and BE using the genome wide association summary statistics of five neuropsychiatric disorders.[8] The GWAS statistics were obtained from the Psychiatric Genomics Consortium (https://www.med.unc.edu/pgc). The study subjects included a total of 27,888 controls and 33,332 cases with either autism spectrum disorder (ASD), attention deficit hyperactivity disorder (ADHD), bipolar disorder (BIP), major depressive disorder (MDD), or schizophrenia (SCZ). All subjects were of European ancestry and no overlapping subject existed across the five studies. Summary statistics for each disorder included about 1.2 million SNPs obtained from imputation using HapMap III data. In our evaluation, we included 1,105,533 SNPs with imputation quality score *R*^2^ ≥ 0.6 and minor allele frequency ≥ 1% in all five studies. The original cross disorder study identified four genome-wide significant loci with pleiotropic effects using the inverse-variance-weighted fixed-effects meta-analysis method.[8] For the three methods—ASSET, CPASSOC, and BE—we performed meta-analyses of the five disorders and compared the nominated lists of associated traits with those reported in the original publication using log-linear modeling[27] of individual-level genotype data.

### Investigation of study correlations due to overlapping samples

To examine how each method deals with sample overlaps, we used the GWAS data of individuals with doctor-diagnosed asthma or allergic diseases obtained from the UK Biobank (data-field 6152)). Among 152,249 participants with genotype data, a total of 112,551 of European ancestry with high-quality genotyping and complete phenotype/covariate data were used in this study. After excluding subjects with both allergic diseases and asthma, the study sample included 19,508 subjects with allergic diseases (hay fever/allergic rhinitis or eczema), 7,908 subjects with asthma, and 76,768 controls without the two conditions. Detailed genotyping and QC procedures of UK Biobank were described previously (http://biobank.ctsu.ox.ac.uk/) and elsewhere.[28] After standard QC procedures, the imputed genotype dosage data contained 7,489,529 SNPs. For each disorder, we performed three genome-wide association studies with the same cases but varying the number of overlapping controls between the two disorder datasets from 0%, to 50%, and to 100%. First, for the no overlapping scenario (0%), 76,768 controls were randomly divided into two groups A and B, each with 38,384 individuals. Subjects in A and B were used as controls in the association study of allergic diseases and asthma, respectively. Secondly, for the 50% overlapping scenario, 76,768 controls were randomly divided into three groups, each with 25,586 individuals. The first two groups were combined and referred to as A, while the second and third groups were combined as B, yielding a half of individuals in A and B are shared. Subjects in A and B were used as controls in the association study of allergic diseases and asthma, respectively. Thirdly, for the 100% overlapping scenario, all 76,768 controls were used in the association study of both disorders. To assess association of an SNP variant with each disorder, additive logistic regression was conducted, adjusting for age, sex, genotyping array, and ten ancestry principal components using PLINK.[29] After meta-analysis of the two GWAS, the numbers of genome-wide significant loci (*P*_*meta*_ ≤ 5 x 10^−8^) were compared among FEMA, ASSET, CPASSOC, and WISC.

We also examined the type I error rates occurring from overlapping samples for the four methods FEMA, ASSET, CPASSOC, and WISC. Using 120,000 samples from the UK Biobank data, we generated two artificial GWAS samples, each with randomly selected 30,000 cases. The remaining 60,000 subjects were splitted into the two GWAS samples as controls by varying the percentage of their overlapping (0%, 50%, and 100%) as described in the previous section. Meta-analyses of the three pairs of GWAS samples were performed using FEMA, ASSET, CPASSOC, and WISC. We repeated this procedure 1,000 times, and counted the proportion of meta-analysis results where genome-wide significant loci were identified (*P*_*meta*_ ≤ 5 x 10^−8^). Note that in all settings, simulated GWAS samples A and B have no heritability due to a random assignment of phenotypes.

## Results

### Discovery of pleiotropic effects in the presence of heterogeneity

**Fig 1:**
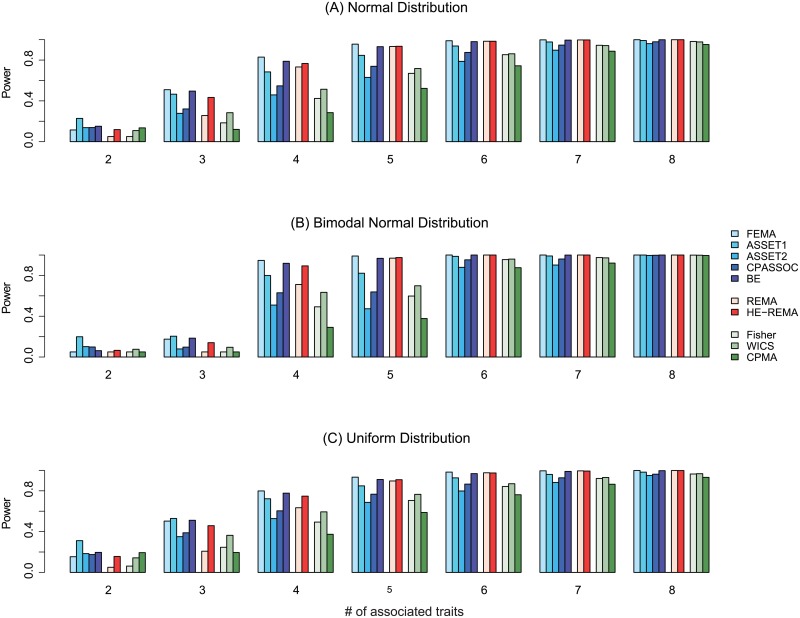
Power (K = 8, all effects in the same direction). In each simulation, a total of 10,000 summary association statistics were generated for eight traits. The numbers of subjects were 1,000 cases and 1,000 controls. The minor allele frequency (MAF) of each causal variant was 0.1. The three panels from the top to the bottom represent when the effect of the variant was drawn from a normal distribution, a bimodal normal distribution, and a uniform distribution. In each graph, the Y-axis denotes the power of each method at an alpha level of 0.05 while the X-axis shows the number of truly associated traits. All associated traits shared the effects in the same direction. Ten meta-analysis methods were separated into three groups each based on: (1) the fixed-effects model (blue hue), (2) the random-effects model (red hue), and (3) the p-value-based model (green hue).

We first investigated the type I error and power of ten meta-analysis methods in the presence of various heterogeneous effects. Specifically, we considered three study settings: (1) different distribution models for effect sizes, (2) a subset of traits with null effects, and (3) existence of opposite directional effects. Fig 1 summarizes the power of ten meta-analysis methods we evaluated assuming that all associated variants carry effects in the same direction across eight study traits (S2–S13 Figs for five and ten traits under various simulation settings). Panels (A), (B), and (C) illustrate the results under the normal, bimodal normal, and uniform distributions of effect sizes, respectively. For clarity, we grouped the ten methods we evaluated into three groups. The first group includes five methods based on the fixed-effects model: FEMA, ASSET1, ASSET2, CPASSOC, and BE. The second group includes two methods based on the random-effects model: REMA and HE-REMA. The third group includes three methods that utilize *p*-value-based integration: Fisher, WICS, and CPMA. The Y-axis shows the power of each method, while the X-axis displays the number of associated traits varying from one to five in the order of decreased heterogeneity.

The simulation results showed that the ASSET1 method, which conducts an exhaustive search of all subset-based fixed-effects models, performs the best when less than a half of traits carry effects. As the number of associated traits increased, ASSET1 however lost its edge, revealing the substantial burden of multiple testing corrections arising from its exhaustive search. In contrast, FEMA, BE, and HE-REMA showed much improved power when a variant is associated with more than a half of traits (Fig 1). It is notable that the classic fixed-effects model, FEMA, featured the highest performance under various heterogeneous scenarios we tested along with the increased number of traits with non-null effects. P-value-based methods show in general inferior performance to the methods based on random or fixed-effects-based models.

**Fig 2:**
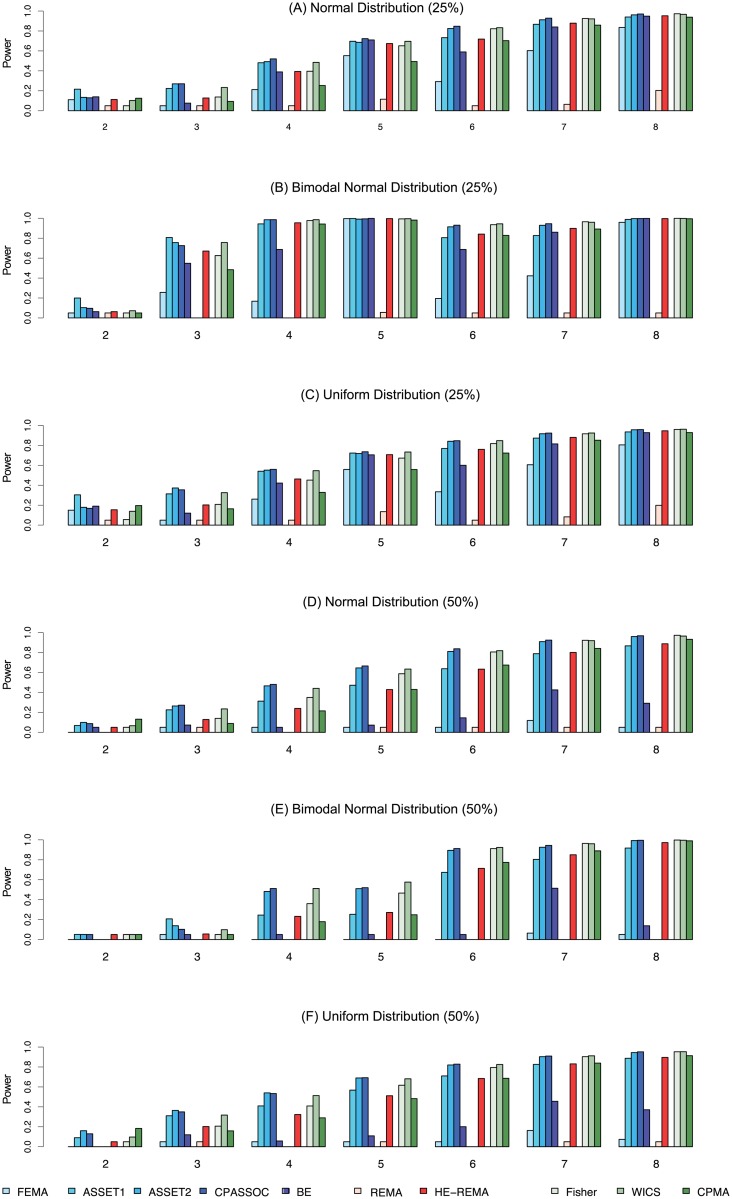
Power (K = 8, some effects in the opposite direction). The same simulation was conducted as previously except that the direction of some effects was opposite. The three panels (A), (B), and (C) from the top to the bottom represent when 25% of associated traits carry effects in the opposite direction and effect sizes were drawn from a normal distribution, a bimodal normal distribution, and a uniform distribution, respectively. In each graph, the Y-axis denotes the power of each method at an alpha level of 0.05 while the X-axis shows the number of truly associated traits. The panels (D), (E), and (F) summarize the power of the ten methods when 50% of effects are in opposite direction.

Fig 2 illustrates the simulation results when some of associated traits include opposite directional effects. The three panels (A), (B), and (C) on the left side represent the power of the ten methods under three effect distributions when 25% of effects are in the opposite direction. The panels (D), (E), and (F) shows the same results when 50% of traits carry effects in the opposite direction. In many settings, we found CPASSOC shows the best performance among the compared methods. The advanced performance of CPASSOC was somewhat expected as the method is agnostic to the direction of the effects. The ASSET2 method, which examines all association models including opposite directional effects, followed CPASSOC by a narrow margin, revealing again the substantial burden of its multiple testing corrections. P-value-based meta-analysis methods do not take into account the direction of effects, displaying much enhanced power in this setting; the WICS method in particular showed comparable power to CPASSOC or ASSET2 in multiple cases. The classic random-effects model, REMA showed the least performance, while HE-REMA demonstrated noticeably improved power compared to REMA. The relative performance of the ten methods was largely stable under different distributions (normal, bimodal normal, and uniform), sizes of effects (Odds Ratio of 1.1, 1.2), trait numbers (*K* = 5, 8, 10), and significance levels (α = 0.05, 0.001). S2–S13 Figs display the performance results under major simulation settings.

We also examined the estimated type I error rates of the ten meta-analysis methods. S1 Table summarizes the results based on 10,000 simulated datasets with only null effects. All methods provided well controlled type I error rates at the tested significance levels of α = 0.05, 0.01, and 0.001.

### Characterization of pleiotropic effects

We next evaluated the performance of four meta-analysis methods that prioritize a subset of study traits with which a genetic variant is most likely to be associated: ASSET1, ASSET2, CPASSOC, and BE. Table 2 summarizes the sensitivity (i.e., the average proportion of detected associated traits) and the specificity (i.e., the average proportion of discarded null traits) calculated over 10,000 replicates in each simulation setting under the bimodal normal distribution (S2 Table for all three distributions). When all effects occur in the same direction, the BE_m.0.5/0.5_ method exhibited the best sensitivity, followed by ASSET1/2, CPASSOC, and the conservative *m*-value threshold model BE_m.0.9/0.1_. All methods tended to show improved sensitivity as more traits show non-null effects, but CPASSOC disposed of an opposite trend. When some effects occur in the opposite direction, ASSET2 showed superior sensitivity to other methods in all conditions. ASSET1 featured the best specificity while the other methods showed comparable performance in multiple settings. The BE_m.0.9/0.1_ approach assigned a considerable number of traits to an ambiguous group (0.1 < m-value < 0.9), resulting in a substantial loss of its prediction accuracy.

**Table 2:** Sensitivity and specificity of ASSET, BE, and CPASSOC for predicting trait-specific association.

Bimodal normal	Sensitivity					Specificity				
	ASSET1	ASSET2	BE(m> = 0.9)	BE(m>0.5)	CPASSOC	ASSET1	ASSET2	BE(m< = 0.1)	BE(m< = 0.5)	CPASSOC
**Ta**	**100% effects in the same direction**									
3	0.34	0.335	0.21	0.374	0.326	1	1	0.127	0.907	0.999
4	0.539	0.514	0.259	0.704	0.289	1	1	0.069	0.972	1
5	0.479	0.422	0.235	0.792	0.264	1	1	0.032	0.892	0.999
6	0.554	0.535	0.406	0.866	0.262	1	1	0.033	0.816	0.999
7	0.51	0.49	0.376	0.929	0.269	1	1	0.011	0.64	0.994
8	0.542	0.541	0.477	0.972	0.214	NA	NA	NA	NA	NA
**Ta**	**75% effects in the same direction**									
3	0.38	0.707	0.331	0.348	0.433	1	0.973	0.896	0.995	1
4	0.322	0.658	0.187	0.287	0.433	1	1	0.785	0.999	1
5	0.457	0.656	0.367	0.464	0.353	1	0.975	0.799	1	1
6	0.356	0.59	0.161	0.337	0.275	1	0.999	0.265	0.988	0.999
7	0.336	0.546	0.131	0.325	0.29	1	0.999	0.135	0.981	0.993
8	0.413	0.579	0.256	0.491	0.229	NA	NA	NA	NA	NA
**Ta**	**50% effects in the same direction**									
3	0.337	0.669	0.317	0.32	0.33	1	0.887	0.671	0.956	0.999
4	0.251	0.573	0.25	0.25	0.289	1	0.999	0.941	1	1
5	0.202	0.538	0.2	0.2	0.251	1	0.998	0.667	1	0.998
6	0.308	0.619	0.292	0.331	0.304	1	0.998	0.741	1	0.999
7	0.304	0.578	0.167	0.287	0.275	1	0.998	0.425	0.998	0.994
8	0.266	0.593	0.216	0.251	0.244	NA	NA	NA	NA	NA

T_a_: the number of associated traits

We also investigated the prediction performance of the three meta-analysis methods ASSET1, BE, and CPASSOC using actual GWAS summary statistics of five neuropsychiatric disorders.[8] In terms of the detected number of genome-wide significant loci, the three methods showed comparable power with FEMA, the meta-analysis method that was employed in the original publication.[8] None of the methods showed indication of genomic inflation (QQ and Manhattan plots in S14 and S15 Figs). In sum, we identified a total of six LD-independent regions with genome-wide significance (*P*_meta_ ≤ 5 x 10^−8^; LD-clumping—clump-p1 5e-8—clump-p2 1e-5—clump-r2 0.2—clump-kb 500; S3 Table). These loci include four regions reported in the original publication by the FEMA—3p21.1, 10q24.32, 12p13.33, and 10p12.33—and two regions on 10q21.2 and 6p21.33 additionally detected by ASSET1, ASSET2, BE, and CPASSOC. In the original study,[8] log-linear modeling of pooled genotype data[27] were used to characterize disorder-specific association models for the top GWAS loci. We compared the reported results with those of ASSET1, ASSET2, BE, and CPASSOC in Table 3. All methods unanimously chose the region 10q21.2 as a risk locus specific to bipolar disorder. However, for the other loci, nominated disorder models by each meta-analysis method differed in multiple cases. Log linear modeling and the BE method tend to select parsimonious models, while the ASSET1 method predicts broader effects for the same loci. However, the loci 10q24.32 and 12p13.33 are not predicted by ASSET2.

**Table 3:** Characterization of disorder-specific association for the top genome-wide significant loci from the meta-analysis of five neuropsychiatric disorders.

Index SNP		rs2535629	rs11191454	rs1024582	rs2799573	rs10994359	rs2517614
**Locus**		3p21.1	10q24.32	12p13.33	10p12.33	10q21.2	6p21.33
**Heterogeneity (I2)**		low (22.86)	low (15.26)	considerable (72.52)	low (0)	high (89.60)	high (81.10)
**Associated Genes**		many	many	CACNA1C	CACNB2	ANK3	many
**Prioritized Lists of Associated Disorders**	**Log-linear modeling**	BIP/MDD/SCZ	BIP/MDD/SCZ	BIP/SCZ	ADHD/BIP/MDD/SCZ	BIP	MDD/SCZ
	**ASSET1**	ADHD/ASD/BIP/MDD/SCZ	ADHD/ASD/BIP/MDD/SCZ	ADHD/BIP/SCZ	ADHD/ASD/BIP/MDD/SCZ	BIP	BIP/MDD/SCZ
	**ASSET 2**	ADHD/ASD/BIP/MDD/SCZ	None	None	ADHD/ASD/BIP/MDD/SCZ	BIP	BIP/MDD/SCZ
	**BE**	BIP/MDD/SCZ	BIP/MDD/SCZ	BIP/SCZ	ADHD/BIP/MDD/SCZ	BIP	MDD/SCZ
	**CPASSOC**	ADHD/ASD/BIP/MDD/SCZ	ADHD/ASD/BIP/MDD/SCZ	BIP	ADHD/ASD/BIP/MDD/SCZ	BIP	ADHD/BIP/MDD/SCZ

ADHD: attention deficits hyperactivity disorder; ASD: autism spectrum disorders; BIP: bipolar disorder; MDD: major depressive disorder; SCZ: schizophrenia;

After the publication of the original meta-analysis study,[8] larger datasets of ADHD (N = 20,183 cases and 35,191 controls),[30] ASD (N = 16,539 cases and 157,234 controls),[31] BIP (N = 13,902 cases and 19,279 controls),[32] and SCZ (N = 36,989 cases and 113,075 controls)[33] became available. We thus examined whether the association of the top six loci are replicated in the expanded datasets. Except for the 10q21.2 region predicted as a specific BIP risk locus, we confirmed that all five pleiotropic loci retained or strengthened their association. Moreover, the gene-rich region on 10q24.32, *CACNA1C* on 12p13.33, and the MHC region on 6p21.33 feature a substantially increased level of pleiotropic association.

### Impact of overlapping subjects in the meta-analysis

When studying related traits or disease phenotypes, it is not uncommon to encounter the use of overlapping subjects across different GWAS datasets. Among the ten methods studied here, ASSET, CPASSOC, and WICS are best suited for this situation. We empirically investigated the power of the three methods using GWAS summary statistics of two traits, allergy and asthma, that share genuine genetic correlation (r_g_ = 0.55; *P* = 9.07x10^-13^).[28] While the cases in each dataset stayed the same, we varied the number of overlapping controls between the two datasets to 0%, 50%, and 100%.

**Fig 3:**
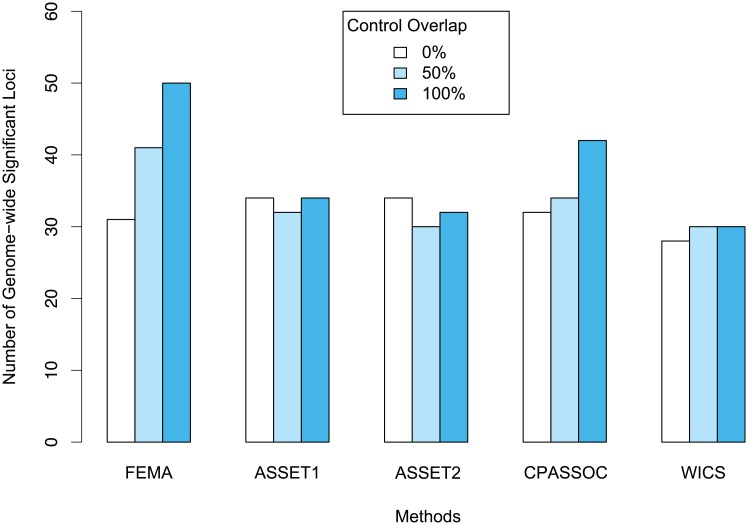
Meta analysis results of GWAS data with shared controls. Three control sample overlapping scenarios (0%, 50%, 100% overlapping) were set up to be used in the association study of both disorders. Total of four methods (ASSET1, ASSET2, CPASSOC, WICS) that are able to control overlapping samples between studies were compared with standard fixed-effect method (FEMA) for their performance in this analysis. The Y-axis denotes number of genome–wide significant loci.

Fig 3 shows the numbers of genome-wide significant loci detected by the four methods—ASSET1/2, CPASSOC, and WICS—along with those of the classic fixed-effects model, FEMA. When two GWAS datasets were independent from each other (i.e., sharing no overlapping subjects), ASSET1 and ASSET2 identified the highest number of genome-wide significant loci (N = 35), followed by CPASSOC (N = 33), FEMA (N = 32), and WICS (N = 29). When controls were shared between the two GWAS, it was apparent that FEMA, which does not take into account artificially induced correlation due to shared subjects, produced markedly increased numbers of genome-wide significant loci that were proportional to the levels of overlapping subjects between the datasets (N = 32 with 0% shared controls; N = 42 with 50% shared controls; N = 51 with 100% shared controls). CPASSOC, which adjusts potential correlation between studies using GWAS summary statistics, also showed an inflated number of genome-wide significant loci proportional to the level of overlapping controls (N = 33 with 0% shared controls; N = 35 with 50% shared controls; N = 43 with 100% shared controls). In contrast, WICS and ASSET presented a similar number of genome-wide significant association findings regardless of the level of shared subjects: ASSET (N = 33~35) and WICS (N = 29~31).

Using simulations, we also examined how the four methods—ASSET1, ASSET2, CPASSOC, and WICS—control falsely induced correlations when two arbitrary traits with no genetic correlation share study samples. S4 Table summarizes the analysis results under three levels of overlapping controls (0%, 50%, and 100%). As with the previous meta-analysis of asthma and allergy, the FEMA, which does not control overlapping controls, produced a markedly increased level of false positive findings in proportion to the raised level of shared subjects; false positive rates were 27% when a half of controls are shared, and the rates rose to 65% when 100% of controls are shared. In contrast, ASSET, CPASSOC, and WICS showed properly controlled rates of type I errors regardless of the proportion of shared controls.

## Discussion

Cross-phenotype GWAS have opened a wide field of genomics research in pleiotropy, but little attention has been put into the evaluation and comparison of analytic methods used in the field. In this study, we aimed to fill this gap by evaluating the performance and the properties of ten meta-analysis methods for GWAS, especially in the context of cross-phenotype studies.

Extensive simulation indicates that the selection of proper analytic methods has important effects on the statistical power and inference of the study findings in cross-phenotype GWAS. Unlike a traditional meta-analysis of a single trait, heterogeneity of genetic effect is inevitable in cross-phenotype GWAS and thus calls into question the use of the fixed-effects model, the most commonly used meta-analysis method in GWAS. In practice, we found that despite the assumption of constant effect sizes, fixed-effects-based methods consistently outperformed the other methods under the presence of diverse heterogeneity. The superior power of the fixed-effects method has previously shown for GWAS meta-analysis of a single trait,[34] which explains its popular use in the field even when the assumption of the constant effect is not appropriate. Our study shows for the first time that even with the existence of multiple traits with null effects, the fixed-effects approach had the largest power. The improved performance of the fixed-effects model in GWAS could also be explained in part because genetic effect sizes are typically very modest (as we employed in our simulation) [35,36]. P-value-based meta-analysis methods provide an alternative strategy to detecting heterogeneous or antagonistic effects but have often been avoided due to a lack of the summary estimate of effect sizes. Our study also indicates that the power of p-value-based integration methods we examined is in general not superior to other methods. It is though clear that when effect sizes of individual studies are not available, the use of p-value-based meta-analysis is inevitable. Further work is warranted to evaluate more p-value-based meta-analysis methods [37–39] in the context of cross-phenotype GWAS.

Characterizing trait-specific association is another important issue in cross-phenotype GWAS because most meta-analysis methods do not nominate specific traits that drive the pleiotropic signal. Manual examination of forest plots is thus often necessary in order to clarify trait-specific association. Three fixed-effects-based methods—ASSET, BE, and CPASSOC—address this limitation by prioritizing the list of most likely associated traits via a model selection procedure. In contrast to the similar level of specificity, sensitivity of the methods varied substantially depending on the direction and the distribution of non-null effects, signifying the need for further method development.

We also investigated how meta-analysis methods deal with artificially inflated correlation among study traits due to overlapping subjects. When individual-level genotype data are available, overlapping subjects are typically split across different studies, making each GWAS independent from each other. Here we looked into an alternative approach that statistically adjusts artificially inflated correlation within the meta-analysis framework. Given the exact numbers of overlapping samples, we confirmed that ASSET and WICS can properly accommodate artificially induced correlation. If such information is not available, CPASSOC is the only usable method, but our study shows that when traits with genuine correlation are meta-analyzed, CPASSOC’s empirical adjustment strategy may not adequately control increasing false positive findings. Caution is thus warranted for the use of CPASSOC in the cross-phenotype studies of potentially overlapping samples.

While we found the superior performance and utility of three fixed-effects-based methods, ASSET, BE, and CPASSOC (as summarized in Table 4), it is clear that several advances are crucial in further methods development. Ideally, the new methods should allow for effect heterogeneity (including the identification of opposite direction allelic effects) and existence of subset-specific association while providing greater power than currently available methods. Another key improvement will be to account for possibly unknown sample overlap between studies, which may lead to potential false positives. A recently proposed method called MTAG [40] describes a promising strategy to address the problem by using bivariate linkage disequilibrium (LD) score regression. The method is however applicable when *all* variants share the same genetic correlation across all traits (i.e., no subset specific effect is assumed), which is violated in most circumstances of cross-phenotype studies. In addition, misleading results can arise when using inadequately fitted meta-analysis models, thus it is recommended to perform the goodness-of-fit test before conducting meta-analysis [41]. Finally, integration of external genomic information in cross-phenotype meta-analysis is a largely unexplored territory. We note that the posterior probability framework on which BE is based has the advantage that flexible priors can be used to incorporate various functional information, such as biological impacts of mutations. Little effort however has been put into exploring the direction. We anticipate further method developments that address these limitations will enable a more in-depth investigation of pleiotropy and its implications for genomic medicine.

**Table 4:** Summary of performance comparison for top fixed-effects models.

Methods	Comparative Power	Control of Type I Error	Control of Overlapping	Sensitivity	Specificity
ASSET	best when T_a_ is small	adequate	adequate	best when opposite directional effects exist	best under most settings
BE	performs well when T_a_ is not small	adequate	adequate	depends on m-value thresholds	depends on m-value thresholds
CPASSOC	best when opposite directional effects exist	adequate	Caution needed when traits with significant genetic correlation are analyzed	intermediate	comparable to ASSET in many settings

T_a_: number of traits with non-null effects;

## Supporting information

S1 Fig Three distributions of effect sizes under odds ratios of 1.1 and 1.2. (PDF)

S2 Fig Power of ten meta analysis methods for five traits when all effects are in the same direction (alpha = 0.05, OR = 1.1, K = 5). (PDF)

S3 Fig Power of ten meta analysis methods for five traits when 25% effects are in the opposite direction (alpha = 0.05, OR = 1.1, K = 5). (PDF)

S4 Fig Power of ten meta analysis methods for five traits when 50% effects are in the opposite direction (alpha = 0.05, OR = 1.1, K = 5). (PDF)

S5 Fig Power of ten meta analysis methods for ten traits when all effects are in the same direction (alpha = 0.05, OR = 1.1, K = 10). (PDF)

S6 Fig Power of ten meta analysis methods for ten traits when 25% effects are in the opposite direction (alpha = 0.05, OR = 1.1, K = 10). (PDF)

S7 Fig Power of ten meta analysis methods for ten traits when 50% effects are in the opposite direction (alpha = 0.05, OR = 1.1, K = 10). (PDF)

S8 Fig Power of ten meta analysis methods for five traits when all effects are in the same direction (alpha = 0.001, OR = 1.2, K = 5). (PDF)

S9 Fig Power of ten meta analysis methods for five traits when 25% effects are in the opposite direction (alpha = 0.001, OR = 1.2, K = 5). (PDF)

S10 Fig Power of ten meta analysis methods for five traits when 50% effects are in the opposite direction (alpha = 0.001, OR = 1.2, K = 5). (PDF)

S11 Fig Power of ten meta analysis methods for ten traits when all effects are in the same direction (alpha = 0.001, OR = 1.2, K = 10). (PDF)

S12 Fig Power of ten meta analysis methods for ten traits when 25% effects are in the opposite direction (alpha = 0.001, OR = 1.2, K = 10). (PDF)

S13 Fig Power of ten meta analysis methods for ten traits when 50% effects are in the opposite direction (alpha = 0.001, OR = 1.2, K = 10). (PDF)

S14 Fig QQ plots of meta analysis results for five neuropsychiatric disorders. (PDF)

S15 Fig Manhattan plots of meta analysis results for five neuropsychiatric disorders. (PDF)

S1 Note Summary of evaluated methods. (PDF)

S1 Table Type I error rates of ten meta analysis methods. (XLSX)

S2 Table Sensitivity and specificity of meta analysis methods, ASSET, BE, and CPASSOC. (XLSX)

S3 Table Details of genome-wide significant loci identified by ten meta-analysis methods. (XLSX)

S4 Table False positive rates of ASSET, CPASSOC, and WICS with overlapping samples. (XLSX)
