# Supplementary figures and images for: Statistical power and utility of meta-analysis methods for cross-phenotype genome-wide association studies
Source: PLoS One. 2018 Mar 1;13(3):e0193256. doi: 10.1371/journal.pone.0193256 (PMC5832233; doi:10.1371/journal.pone.0193256)

Figure S1. Distributions of Effect Sizes

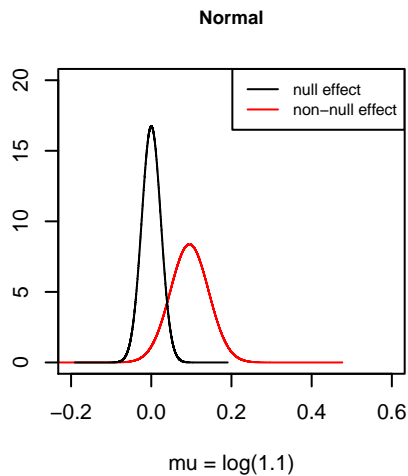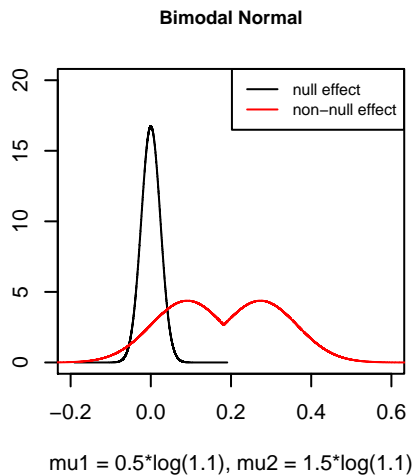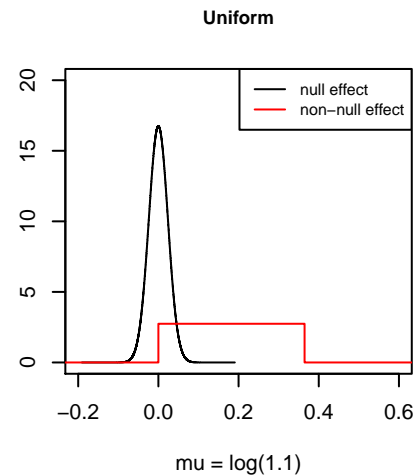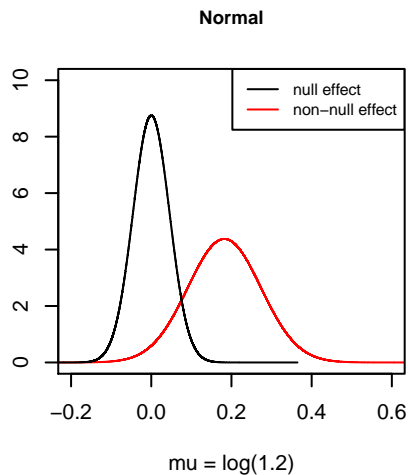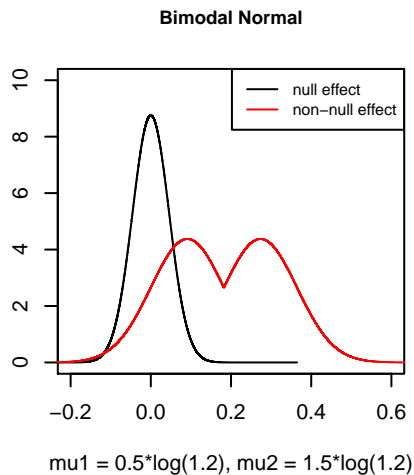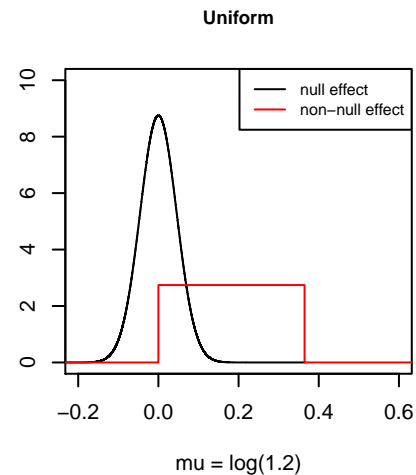

Supplement: S1 Fig — (PDF) [file pone.0193256.s001.pdf]

Figure S14. QQ Plots

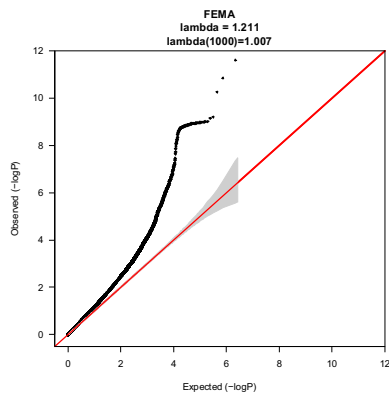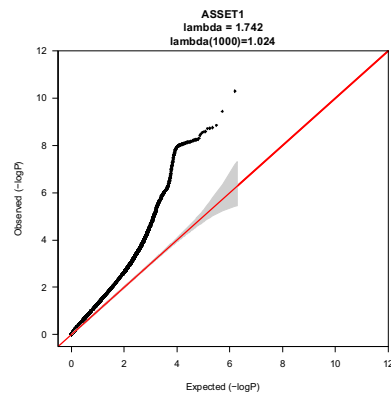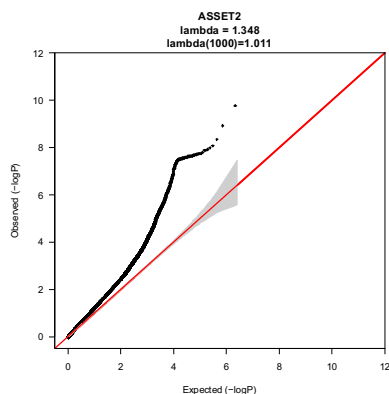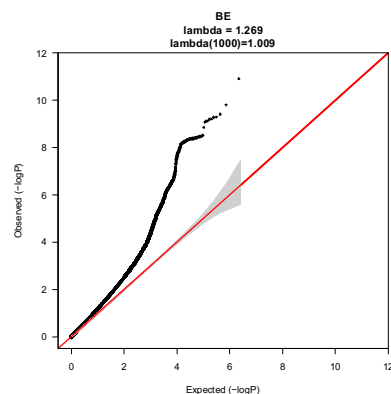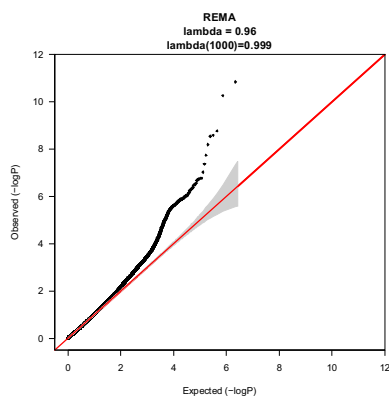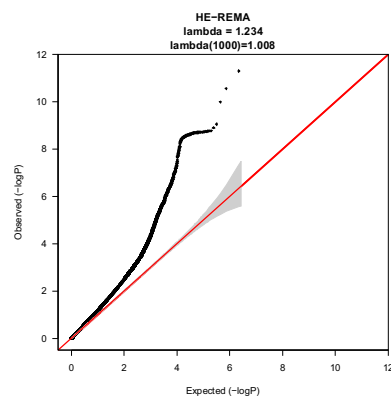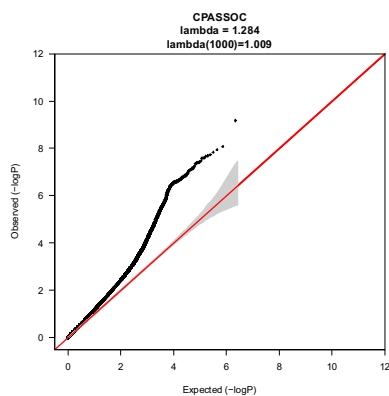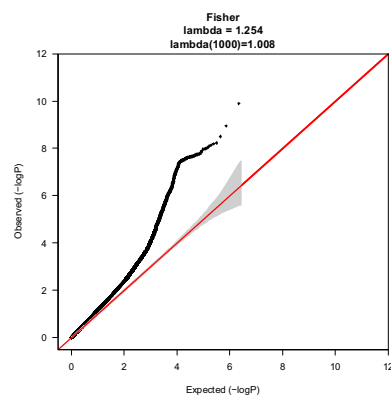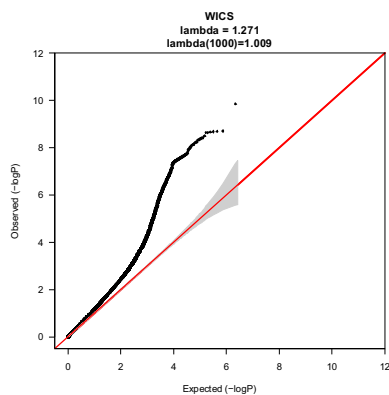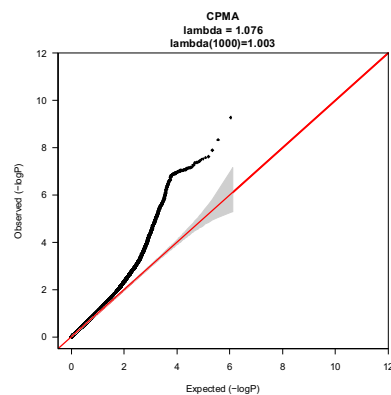

Supplement: S14 Fig — (PDF) [file pone.0193256.s014.pdf]

Figure S15. Manhattan Plots

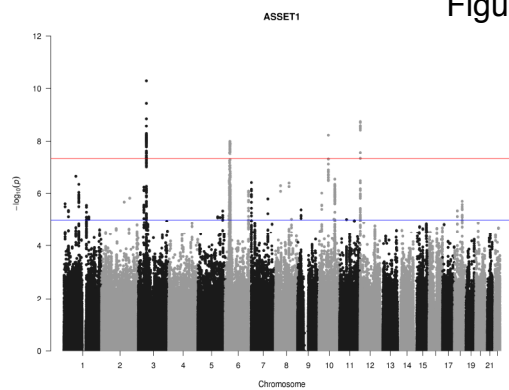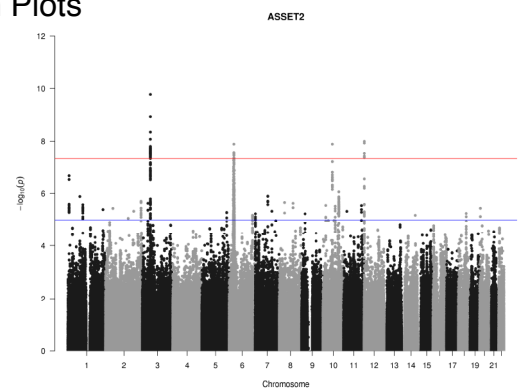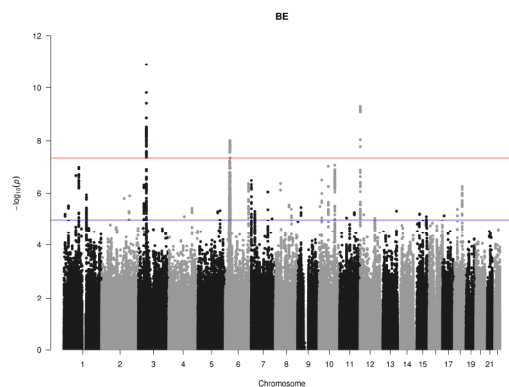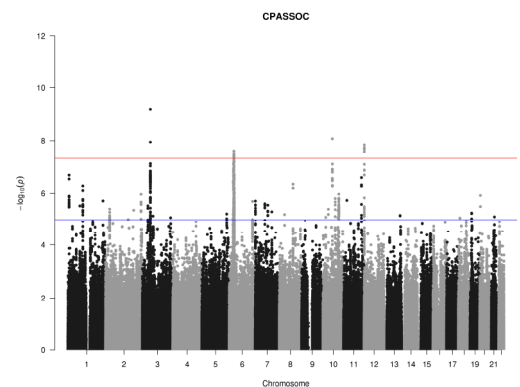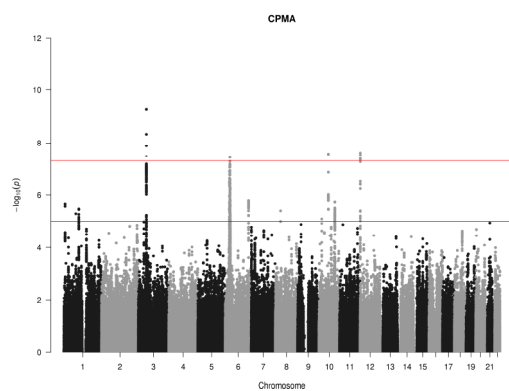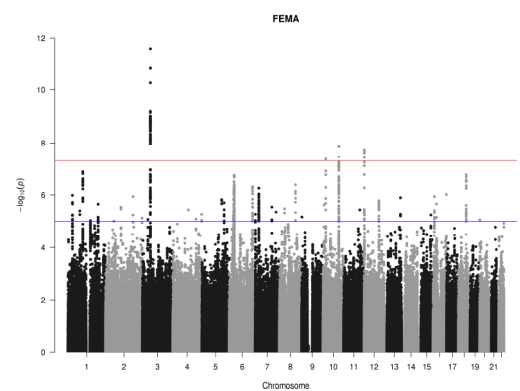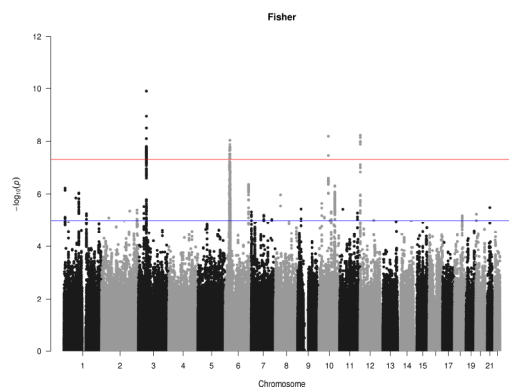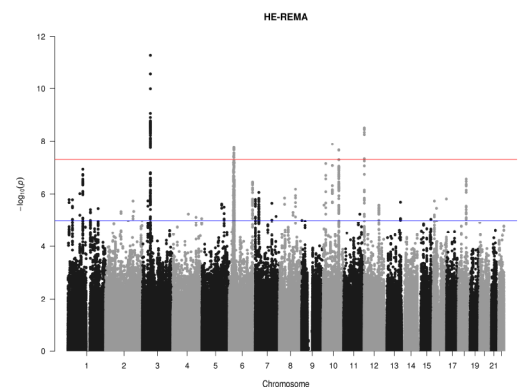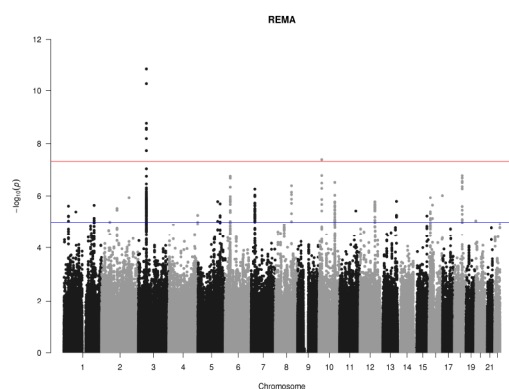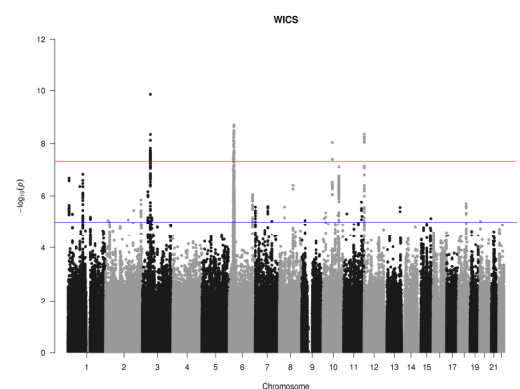

Supplement: S15 Fig — (PDF) [file pone.0193256.s015.pdf]
