# Supplementary material for: Statistical power and utility of meta-analysis methods for cross-phenotype genome-wide association studies
Source: PLoS One. 2018 Mar 1;13(3):e0193256. doi: 10.1371/journal.pone.0193256 (PMC5832233; doi:10.1371/journal.pone.0193256)
Supplement: S3 Fig — (PDF) [file pone.0193256.s003.pdf]

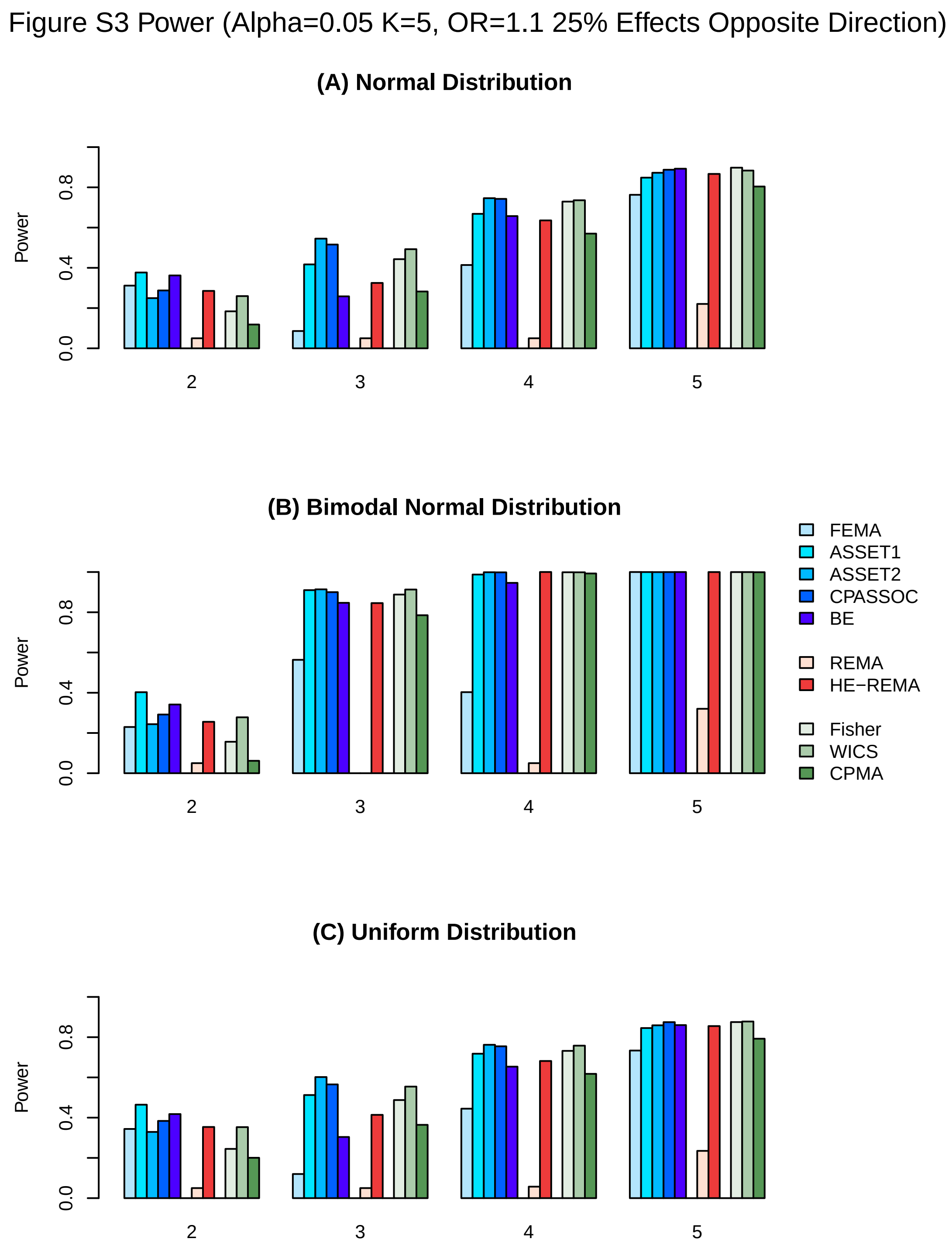
Figure S3 Power (Alpha=0.05 K=5, OR=1.1 25% Effects Opposite Direction)
(A) Normal Distribution
(B) Bimodal Normal Distribution
(C) Uniform Distribution
Power
0.0
0.4
0.8
2
3
4
5
FEMA
ASSET1
ASSET2
CPASSOC
BE
REMA
HE−REMA
Fisher
WICS
CPMA
