# Supplementary material for: Statistical power and utility of meta-analysis methods for cross-phenotype genome-wide association studies
Source: PLoS One. 2018 Mar 1;13(3):e0193256. doi: 10.1371/journal.pone.0193256 (PMC5832233; doi:10.1371/journal.pone.0193256)

Figure S11 Power (Alpha=0.001 K=10, OR=1.2 All Effects Same Direction)

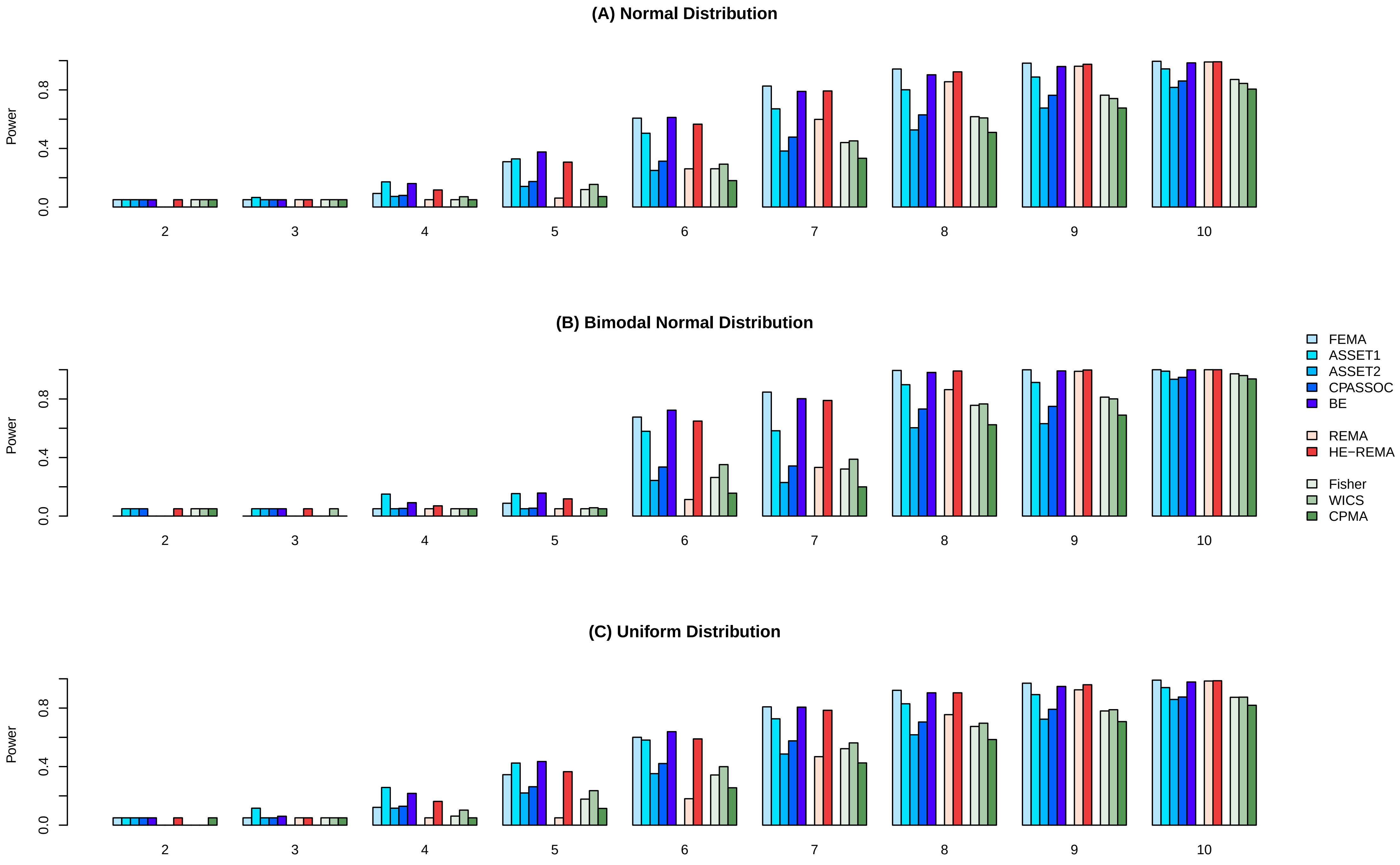

Supplement: S11 Fig — (PDF) [file pone.0193256.s011.pdf]
