# Supplementary material for: Statistical power and utility of meta-analysis methods for cross-phenotype genome-wide association studies
Source: PLoS One. 2018 Mar 1;13(3):e0193256. doi: 10.1371/journal.pone.0193256 (PMC5832233; doi:10.1371/journal.pone.0193256)
Supplement: S1 Note — (PDF) [file pone.0193256.s016.pdf]

Supplementary Note S1. Summary of evaluated methods

Here we briefly describe distinct features of these methods. Let us first define some common notations. We consider a study of *K* phenotypes. For a variant in each study of a given phenotype *k* (1≤*k*≤*K*), we denote the effective number of study subjects, estimated effect size, and sampling variance as $n_k, X_k, \text{and } se_k^2,$ respectively. The Z-score of a variant for each study is calculated as $Z_k = \frac{X_k}{se_k}$.

FEMA (Fixed-Effects Meta-analysis): Fixed-effect meta-analysis assumes a homogeneous effect size of a genetic variant across study phenotypes. Here we use the inverse-variance weighted method[1] that calculates a mean of the effect sizes while weighing each study using the inverse of the variance. This method is approximately equivalent to the weighted sum-of-*Z*-score model (Z). The Z-score of FEMA is calculated as:

$$\mathrm{Z}_{\mathrm{FEMA}} = \frac{\sum_{k=1}^{K}(w_k X_k)}{\sqrt{\sum_{k=1}^{K} w_k}}$$

where $w_k = \frac{1}{se_k^2}$.

ASSET1/ASSET2: ASSET is a generalized fixed-effects meta-analysis package that examines all possible subsets of study traits for the detection of shared association signals.[2] To correct for multiple testing arising from the exhaustive sub-set-based search, ASSET employs the discrete local maxima (DLM) method that efficiently estimates tail probabilities of the examined *Z*-score test statistic. When *T* represents a

set of study traits selected from *K* studies, meta-analysis statistic of the one-sided test (ASSET1) is defined as:

$$\mathrm{Z}_{\mathrm{ASSET1}} = max_{T \in Powerset(\{1,2,\ldots,K\})} \mid Z(T) \mid \; = max_{T \in Powerset(\{1,2,\ldots,K\})} \left| \sum_{k \in T} \sqrt{\pi_k(T)} Z_k \right|$$

where $\pi_k(T) = n_k / \sum_{k \in T} n_k$ represents the sample size of the study *k* relative to the total sample size of the given subset *T*. ASSET also provides the two-sided test, which we refer to as ASSET2, that allows detection of effects in opposite directions:

$$\mathrm{Z}_{\mathrm{ASSET2}} = Z^{(2)}_{max-meta} = -2[\log \tilde{P}^{+}_{DLM} + \log \tilde{P}^{-}_{DLM}]$$

$$\tilde{P}^{(2)}_{DLM} = P\left(\chi^2_4 > Z^{(2)}_{max-meta}\right)$$

where $\tilde{P}^{+}_{DLM}$ and $\tilde{P}^{-}_{DLM}$ are the conditional p values of the strongest association captured in the subsets of studies in positive and negative directions respectively.

BE (Binary Effects Model): The BE is another fixed-effects-based meta-analysis method that specifically targets the scenarios when only a subset of study traits show an effect.[3] For this purpose, the BE method first calculates a posterior probability *m_k* that indicates whether an effect exists in the study *k*.

$$m_i = P(E_i = 1|Y) = \frac{P(Y|E_i = 1)P(E_i = 1)}{P(Y|E_i = 0)P(E_i = 0) + P(Y|E_i = 1)P(E_i = 1)}$$

$$= \frac{\sum_{e \in U_i} P(Y|E = e)P(E = e)}{\sum_{e \in U} P(Y|E = e)P(E = e)}$$

where U_i is a subset of U whose elements' ith value is 1. The association statistic of the BE model is then calculated by using this pre-estimated posterior probability as a weight while aggregating individual study effects as below:

$$S_{BE} = \frac{\sum m_k \sqrt{w_k} Z_k}{\sqrt{\sum m_k^2 w_k}} \text{ where } w_k = N_k.$$

CPASSOC (Cross-phenotype Association): Similar to the ASSET and BE, CPASSOC assumes that effects may exist only within a subset of study traits.[4] However, unlike those two methods, CPASSOC identifies the subset of studies with effects by sequentially adding a trait by an incremental order of their association significance. Among the sequentially examined subsets, the one with the highest meta statistics is selected. The proposed set-based meta statistics is as follows:

$$S(\tau) = \frac{e^{\tau}\left(R(\tau)W(\tau)\right)^{-1}U(\tau)\left(e^{T}\left(R(\tau)W(\tau)\right)^{-1}U(\tau)\right)^{T}}{e^{T}W(\tau)^{-1}R(\tau)^{-1}W(\tau)^{-1}e}$$

$$S_{Het} = \max_{\tau>0} S(\tau)$$

where $R(\tau)$ is a submatrix of R representing the correlation matrix between study traits, $U(\tau)$ is the sub-vector of the Wald test statistic U satisfying $U_{jk} > \tau$, and $W(\tau)$ is the diagonal submatrix of W, corresponding to $T(\tau)$.

REMA (Random-Effects Meta-analysis): The random-effect meta-analysis model assumes that the mean effect of a variant could vary across different studies, and therefore the variance of the mean distribution represents the variance within studies as well as between-study heterogeneity.[5] The combined effect in the random effects meta-analysis thus represents the mean of the distribution of true effects. Using the method proposed by DerSimonian and Laird,[6] we calculate the Z-score of REMA as below:

$$\mathrm{Z}_{\mathrm{REMA}} = \frac{\sum_{k=1}^{K} w'_k X_k}{\sqrt{\sum_{k=1}^{K} w'_k}} \text{ where}$$

$$w'_k = \frac{1}{\frac{1}{w_k}+\tau^2},\ \ w_k = \frac{1}{se_k^2},\ \ \tau^2 = \frac{Q-(k-1)}{\sum w_k - (\frac{\sum {w_k}^2}{\sum w_k})}, \text{and } Q = \sum w_k (X_k - \bar{X})^2.$$

Here Q is Cochran's Q statistic representing the total variance, while *k-1* represents the expected variance when all studies have the same true effect.

HE-REMA (Han and Eskin's Random Effects Model): The HE-REMA is a newly developed random-effects meta-analysis method that aims to improve the discovery power for heterogeneous effects.[7] Unlike classical REMA, Han and Eskin's model assumes no heterogeneity under the null hypothesis, thus increasing the detection power in the presence of between-study heterogeneity. We used the HE-REMA model implemented in the METASOFT package. The statistic of HE-REMA is defined as:

$$S_{HE-REMA} = S_{FE} + S_{Het} \text{ where}$$

$$S_{FE} = \left\{\sum \frac{X_i^2}{V_i} - \sum \frac{(X_i - \hat{\mu})^2}{V_i}\right\},$$

$$S_{Het} = \left\{\sum log\,(\frac{V_i}{V_i + \hat{\tau}^2} + \sum \frac{(X_i - \hat{\mu})^2}{V_i} - \sum \frac{\left(X_i - \bar{\hat{\mu}}\right)^2}{V_i + \hat{\tau}^2}\right\},$$

and $\bar{\hat{\mu}}$ is the maximum likelihood estimate of the average effect size under the restriction $\tau^2 = 0$.

Fisher (Fisher's Method): Another major category of classic meta-analysis approaches combines evidence of association by aggregating *p*-values, rather than effect sizes, across study traits. Here we test Fisher's method, which is asymptotically optimal and efficient when the combined *p*-values are independent.[8]

$$X^2 = -2\sum_{k=1}^{K} log\,(p_k).$$

Under the null hypothesis and independence of study traits, p-values follow a uniform distribution and the corresponding X^2 statistic approximates a chi-squared distribution with 2*K* df.

WICS (Weighted Inverse Chi-Square): The WICS method calculates the meta-analysis statistic by summing the weighted chi-squares of individual studies. Here we used the WICS method implemented in the R package by Zaykin and Kozbur,[9] which

uses the squared root of the study sample size as a weight. Given study *p*-values, the chi-square statistic of individual studies are obtained from the inverse transformation of the chi-square distribution with 1 df.

$$\chi_i^2 = \Psi^{-1}(1 - p_i)$$

where Ψ^{-1} denotes the inverse chi-square distribution with 1 df. The weights for the underlying multivariate normal scores are given by

$$w_i = \sqrt{N_i(\boldsymbol{R}^{-1})_{ii}}.$$

For k studies, the weighted sum of independent inverse chi-squares is given by

$$S_{WICS} = \sum_{i=1}^{k} w_i^2 \chi_i^2.$$

CPMA (Cross-Phenotype Meta-analysis): The CPMA method examines whether the distribution of observed association *p*-values across multiple traits deviates from the distribution of random *p*-values expected under the null hypothesis.[10] If all study traits are independent and not associated with a genetic variant, the association *p*-values are expected to be uniformly distributed and $-\log(p)$ is exponentially decaying with a decay rate $\lambda = 1$. To measure the discrepancy, the CPMA test statistic $\boldsymbol{S_{CPMA}}$ is defined with a likelihood ratio test as:

$$S_{CPMA} = -2 \times \frac{P(Data|\lambda = 1)}{P(Data|\lambda = \hat{\lambda})}$$

The CPMA *p*-value is obtained by a 1 degree of freedom chi-square test.

Reference

1. Greenland S (1987) Quantitative methods in the review of epidemiologic literature. Epidemiol Rev 9: 1-30.
2. Bhattacharjee S, Rajaraman P, Jacobs KB, Wheeler WA, Melin BS, et al. (2012) A subset-based approach improves power and interpretation for the combined analysis of genetic association studies of heterogeneous traits. Am J Hum Genet 90: 821-835.
3. Han B, Eskin E (2012) Interpreting meta-analyses of genome-wide association studies. PLoS Genet 8: e1002555.
4. Zhu X, Feng T, Tayo BO, Liang J, Young JH, et al. (2015) Meta-analysis of correlated traits via summary statistics from GWASs with an application in hypertension. Am J Hum Genet 96: 21-36.
5. Cochran WG (1954) The Combination of Estimates from Different Experiments. Biometrics 10: 101-129.
6. DerSimonian R, Laird N (1986) Meta-analysis in clinical trials. Control Clin Trials 7: 177-188.
7. Han B, Eskin E (2011) Random-effects model aimed at discovering associations in meta-analysis of genome-wide association studies. Am J Hum Genet 88: 586-598.
8. Fisher RA (1925) Statistical Methods for Research Workers. Edinburgh: Oliver and Boyd.
9. Zaykin DV, Kozbur DO (2010) P-value based analysis for shared controls design in genome-wide association studies. Genet Epidemiol 34: 725-738.
10. Cotsapas C, Voight BF, Rossin E, Lage K, Neale BM, et al. (2011) Pervasive sharing of genetic effects in autoimmune disease. PLoS Genet 7: e1002254.
